# Epidemiology and phylodynamics of multiple clades of H5N1 circulating in domestic duck farms in different production systems in Bangladesh

**DOI:** 10.3389/fpubh.2023.1168613

**Published:** 2023-07-06

**Authors:** Ariful Islam, Mohammad Enayet Hossain, Emama Amin, Shariful Islam, Monjurul Islam, Md Abu Sayeed, Md Mehedi Hasan, Mojnu Miah, Mohammad Mahmudul Hassan, Mohammed Ziaur Rahman, Tahmina Shirin

**Affiliations:** ^1^EcoHealth Alliance, New York, NY, United States; ^2^Centre for Integrative Ecology, School of Life and Environmental Sciences, Deakin University, Geelong, VIC, Australia; ^3^One Health Laboratory, International Centre for Diarrheal Diseases Research, Bangladesh (icddr,b), Dhaka, Bangladesh; ^4^Institute of Epidemiology, Disease Control and Research (IEDCR), Dhaka, Bangladesh; ^5^Queensland Alliance for One Health Sciences, School of Veterinary Science, University of Queensland, Brisbane, QLD, Australia

**Keywords:** avian influenza, HPAI H5N1, waterfowl, risk factors, phylogeny, 2.3.2.1a, 2.3.4.4b, zoonotic

## Abstract

Waterfowl are considered to be natural reservoirs of the avian influenza virus (AIV). However, the dynamics of transmission and evolutionary patterns of AIV and its subtypes within duck farms in Bangladesh remain poorly documented. Hence, a cross-sectional study was conducted in nine districts of Bangladesh between 2019 and 2021, to determine the prevalence of AIV and its subtypes H5 and H9, as well as to identify risk factors and the phylodynamics of H5N1 clades circulating in domestic duck farms. The oropharyngeal and cloacal swab samples were tested for the AIV Matrix gene (M-gene) followed by H5, H7, and H9 subtypes using rRT-PCR. The exploratory analysis was performed to estimate AIV and its subtype prevalence in different production systems, and multivariable logistic regression model was used to identify the risk factors that influence AIV infection in ducks. Bayesian phylogenetic analysis was conducted to generate a maximum clade credibility (MCC) tree and the maximum likelihood method to determine the phylogenetic relationships of the H5N1 viruses isolated from ducks. AIV was detected in 40% (95% CI: 33.0–48.1) of the duck farms. The prevalence of AIV was highest in nomadic ducks (39.8%; 95% CI: 32.9–47.1), followed by commercial ducks (24.6%; 95% CI: 14.5–37.3) and backyard ducks (14.4%; 95% CI: 10.5–19.2). The H5 prevalence was also highest in nomadic ducks (19.4%; 95% CI: 14.0–25.7). The multivariable logistic regression model revealed that ducks from nomadic farms (AOR: 2.4; 95% CI: 1.45–3.93), juvenile (AOR: 2.2; 95% CI: 1.37–3.61), and sick ducks (AOR: 11.59; 95% CI: 4.82–32.44) had a higher risk of AIV. Similarly, the likelihood of H5 detection was higher in sick ducks (AOR: 40.8; 95% CI: 16.3–115.3). Bayesian phylogenetic analysis revealed that H5N1 viruses in ducks belong to two distinct clades, 2.3.2.1a, and 2.3.4.4b. The clade 2.3.2.1a (reassorted) has been evolving silently since 2015 and forming at least nine subgroups based on >90% posterior probability. Notably, clade 2.3.4.4b was introduced in ducks in Bangladesh by the end of the year 2020, which was genetically similar to viruses detected in wild birds in Japan, China, and Africa, indicating migration-associated transmission of an emerging panzootic clade. We recommend continuing AIV surveillance in the duck production system and preventing the intermingling of domestic ducks with migratory waterfowl in wetlands.

## 1. Introduction

The avian influenza virus (AIV) has garnered increased attention recently because of its impact on productivity, commerce, and human health. The highly pathogenic avian influenza (HPAI) H5N1 virus has been linked to poultry epidemics and occasional human infections worldwide (1). In Bangladesh, the epidemic of H5N1 in poultry was reported for the first time in 2007. Since then, the disease has spread throughout the country, with 585 H5N1 outbreaks reported until the end of 2020 (2–4). In contrast, the first human case of H5N1 was detected through exposure to slaughtered poultry in Bangladesh on May 22, 2008 (5). AIV is now considered to be endemic and some recent research has identified a high percentage of AIV in birds from farms and live bird markets (LBM) including peri-urban and rural settings (6–9). Waterfowl from the order of Anseriformes (including ducks, geese, and swans), are distributed worldwide due to aquatic habitats and are considered one of the major natural reservoirs for AIV (10, 11). Other than domestic duck species, migratory waterfowl stopover for a few days to several weeks to rest at foraging areas (wetlands and lakes) along their migratory routes (10, 12). The AIV can spread to and from domestic duck populations due to the length of stay and wetland of both domestic and migratory duck populations, and the asymptomatic nature of infected individuals increases the likelihood that the virus will spread to other species (13). When an infected duck defecates in a specific wetland or waterbody, the AIV enters the environment and infects other ducks easily while they access the same areas. Although AIV has been replicated in the respiratory tract, we cannot overlook the fecal shedding of the AIV (14). Consequently, wetlands and water bodies can become contaminated with AIV through the defecation of infected birds, therefore, transmission of the virus is more likely when a significant number of birds roost on a small wetland (15). This evidence can be corroborated by another study in which the authors recovered the virus from the lake surface, where many different duck species graze (16). So, the high AIV titer in feces, the stability of the virus in the water, and the higher number of positive cloacal than tracheal samples suggest the virus persists in duck populations through fecal-oral transmission (17). Therefore, the present study is conducted to estimate the prevalence and risk factors of AIV in domestic ducks under different rearing systems and landscapes.

Bangladesh is an agriculture-based country where the total livestock population comprises around 311.8 million chickens and 63.85 million ducks throughout the country (18), which are housed in over 53 thousand commercial broiler farms, 18 thousand commercial layer farms, and 6.5 thousand commercial duck farms, whereas in rural settings on an average, each household rears 6.8 chickens and ducks in backyard systems for their consumption or even commercial activity (19, 20). Furthermore, Bangladesh is also known as a riverine country due to its numerous transboundary rivers, suitable habitats, and wetlands that attract millions of migratory birds of 244 species each winter (October to March) and allow them to intermingle with resident aquatic wild birds and domestic ducks (21, 22). Ducks are typically raised for household and commercial production in Bangladesh using nomadic or semi-scavenging systems. Consequently, domestic ducks have frequent access to wetlands and interact closely with various migratory bird species, which may facilitate the evolution and emergence of novel strains of AIV and eventually lead to widespread outbreaks of the virus. The reservoir duck species are able to shed and transmit the virus from the respiratory and intestinal tracts, showing few or no symptoms of the disease. Therefore, understanding the epidemiology of the origin and circulation pattern of H5N1 in the duck population in Bangladesh is deemed a priority.

The AIV RNA prevalence in domestic ducks in parts of Bangladesh has been previously documented as 0.9–89% (23–25), whereas the dominant AIV subtypes were H5 and H9 in ducks (26). Furthermore, since the first detection of HPAI H5N1 viruses, various clades, including 2.2.2, 2.3.2, 2.3.4.2 (27, 28), 2.3.2.1a (29, 30), and 2.3.4.4 (31) clade of H5N6, have been identified in Bangladesh. Besides, the novel reassortant H5N1 clade 2.3.2.1a has already been isolated from the LBMs in Bangladesh, having a close relatedness to the virus isolated from birds sampled in one of the four regions of this country (32). Furthermore, during the last 3 years, clade 2.3.4.4b of the H5N1 virus has recently spread to domestic poultry and wild birds widely in Europe, Africa, Asia, and America, leading to the loss of over 33 million domestic birds (33). On the other hand, northwest Spain encountered an outbreak of 2.3.4.4b H5N1 in Minks (34). Also, this clade of H5N1 was also detected in mammals like harbor porpoises in Sweden (35) and dolphins, Sea lions, Sanderlings, Pelicans, and Cormorants in Peru (36). There have been 893 sporadic human A(H5N1) cases reported from 21 countries since 1997, and eight of those cases have been caused by clade 2.3.4.4b since 2022, which raises the possibility of a pandemic (37, 38). Both nomadic and backyard ducks are reared in a free scavenging system in Bangladesh, sharing open wetlands with large numbers of migratory waterfowl, and other wild birds and transmission of HPAI H5N1 may occur easily where the migratory birds are considered one of the potential routes for introducing new clades of HPAI H5N1 in Bangladesh (39). The surveillance of AIV in ducks from different production systems and patterns of AIV and subtype circulation within these systems are not well documented. Molecular characterization and evolutionary dynamics of HPAI H5N1 in the duck population are crucial. Therefore, we conducted this study to know the prevalence of AIV and their subtypes H5 and H9, risk factors, and phylodynamics of H5N1 clades circulating in domestic ducks in the different production systems in Bangladesh.

## 2. Methodology

### 2.1. Ethical approval

The study protocol was approved by the ethics committee of Chattogram Veterinary and Animal Science University (CVASU) bearing the number CVASU/Dir(R&E) EC/2019/126(1) and CVASU/Dir(R&E)EC/2020/191/7.

### 2.2. Study design and site selection

**Figure 1:**
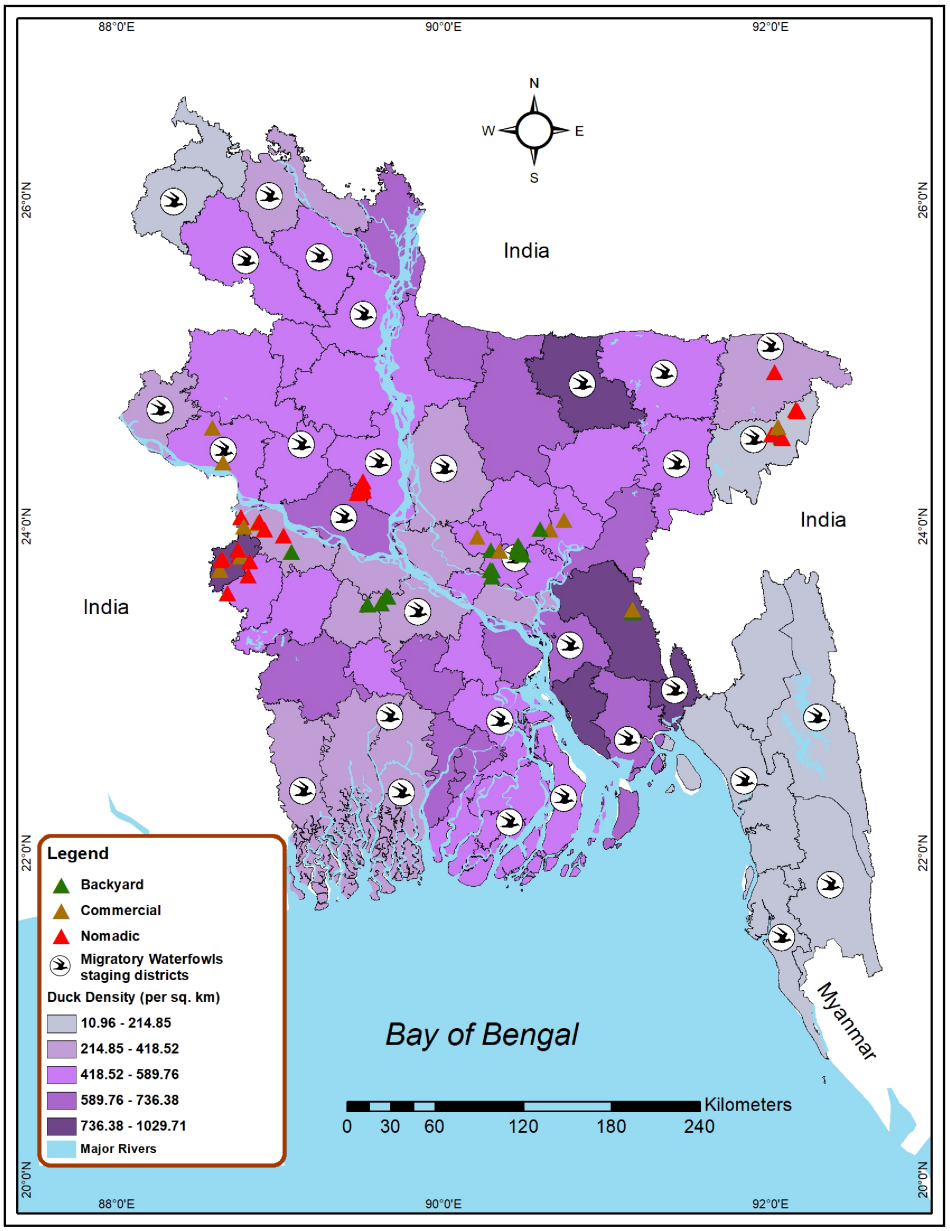
Map locating the site selected for investigating AIV risk analysis among duck farms in Bangladesh (2019–2021). Green triangles represent backyard duck farms, brown triangles represent commercial duck farms, and red triangles represent nomadic duck farms chosen for sampling in this study. Bird symbols denote districts that have migratory bird staging areas. The intensity of the color gradient shows the density of ducks in a district.

Bangladesh currently has three duck production systems: nomadic farms, backyard farms, and commercial farms. Nomadic duck farming is a traditional approach to duck production where the ducks are kept in a free-range habitat and are allowed to roam and feed in various regions (40). In the backyard farming system, household ducks are kept overnight near or within the farmer’s house and travel only over a short distance for scavenging (27), and in the commercial farming system, ducks are kept in total confinement (41). Considering the duck farming patterns, we conducted a cross-sectional study and purposive sampling to find out AIV, H5, and H9 subtype prevalence as well as risk factors among ducks in different production systems from 2019 to 2021 in Bangladesh. The study sites were selected based on duck density, the presence of wetlands, and migrating waterfowl. Data on the distribution of migratory bird staging areas in Bangladesh was obtained from the literature (42, 43). Additionally, the duck density data were gathered from the Bangladesh agriculture census 2019 (44). Figure 1 depicts the nine selected districts of Bangladesh, namely Dhaka, Faridpur, Cumilla, Kushtia, Meherpur, Moulovibazar, Sylhet, Sirajganj, and Rajshahi, which represent the wide spectrum of duck-rearing practices across the country. The sampling of nomadic ducks from Sylhet and Moulovibazar represented the wetland habitats of Haor basin (45), while Kushtia, Meherpur, and Sirajganj were considered as Jamuna floodplains (46). In wetlands, domestic ducks and migratory birds share foraging habits and intermingle. Consequently, backyard ducks were also sampled in wetlands areas. In addition, backyard ducks were sampled from Dhaka, Cumilla, and Faridpur. The samples of commercial duck farms were collected from Cumilla, Dhaka, Kushtia, Meherpur, Rajshahi, and Sylhet (Figure 1).

### 2.3. Sample and data collection procedure

We sampled a total of 522 ducks from 171 farms, with 270 ducks coming from 127 backyard farms, 61 ducks from 11 commercial farms, and 191 from 33 nomadic farms. The samples were collected from both sick and healthy ducks, and common signs observed in sick ducks were torticollis, lack of coordination, leg paralysis, and sudden death, which have also been associated with H5N1 symptoms in previous studies (24, 47). Pooled oropharyngeal with cloacal swabs were collected from each duck by an experienced field veterinarian while causing the birds as little distress as possible. The biological specimens were collected by wearing appropriate personal protective equipment like coveralls, gloves, and other safety equipment. Immediately after sampling, the swabs sticks were placed into a 1.8 ml cryovial containing 1 mL viral transport medium (VTM). Each vial was marked using a unique identification number and placed in the portable dry shipper before transport to the laboratory. In the lab, all the samples were stored at –80°C freezer until further laboratory evaluation. A pre-tested questionnaire and face-to-face interview were used to collect all biosecurity-related data, that could potentially be a risk factor.

### 2.4. Virological testing

The viral RNA was extracted from the pooled swab samples (oropharyngeal and cloacal) using a KingFisher Flex 96-well robot (Thermo Scientific, Waltham, MA) and the MagMAX 96 AI/ND Viral RNA Isolation Kit (Ambion, Inc. Austin, TX) in accordance with the manufacturer’s instructions. Real-time reverse transcriptase PCR (rRT-PCR) was used in conjunction with reference primers and probes to detect the presence of the AIV (InfluA) Matrix (M) gene in viral RNA, as described by the CDC and Spackman (48, 49). Then, InfluA (M-gene) positive samples were examined with specific subtypes primers of H5, H7, and H9 as previously described (49, 50). The samples were considered as AIV positive for the M-gene if the cycle threshold (Ct) was less than 40 and as H5, H7, and H9 positive if Ct < 37 (51). Samples that tested positive for the M gene but negative for H5, H7, and H9 were classified as A/untyped.

### 2.5. H5N1 sequencing

The viral RNA was extracted using QIAamp viral RNA minikit (Qiagen). The influenza segments were amplified following the protocol described by Zhou et al. (52). After amplification, PCR amplicons were visualized by agarose gel electrophoresis, followed by purification in an AMPure XP Bead. Subsequent nanopore sequencing libraries were prepared using Ligation Sequencing Kit (SQK-LSK109) and the Native barcoding approach. In 2019, the Sanger sequencing was deployed to amplify and subjected to partial sequencing of HA and NA genes of the 2 H5N1 virus described by Hoffmann (53). In 2021, the final library was quantified in the Qubit 1× dsDNA High Sensitivity Assay Kit (Invitrogen) with a Qubit 4 fluorometer (Invitrogen) and loaded onto the FLO-MIN106D flow cell on an Oxford Nanopore MinION MK 1C platform. Raw fast5 reads were base called by real-time base-calling with Guppy 4.3.4, released with MinKNOW software with the fast base-calling mode, and subsequent analyses were performed in the appropriate bioinformatics tools. The HA and NA segments of H5N1 sequences were submitted to GenBank under the accession numbers from OQ430759 to OQ430762 and OQ423229 to OQ423237.

### 2.6. Statistical analysis

The frequency, percentage, and univariate value of p were computed at the socio-demographic level of the duck farmer, along with duck-rearing practices in different production systems and landscapes. A descriptive analysis was computed to determine the prevalence of AIV, H5, and H9 according to the different factors at the individual bird and flock levels. The cross-tabulation and chi-square tests were performed to identify the risk factors between AIV and H5N1 with different bird-level risk factors. Furthermore, the risk factors that were determined as significant at univariate analysis were forwarded to multivariable logistic regression. The likelihood ratio (Wald test) with a value of *p* of ≤0.05 was used to identify the primary risk factor. The results were presented as Adjusted Odds Ratios (AOR), 95% confidence intervals, and values of p The data generated from this study were stored in MS Excel 2021 and checked the data integrity in MS Excel. We used RStudio version 4.1.2 for statistical analysis. We used “lme4” and “tidyverse” packages for the analysis in R software. The ArcGIS^1^ software was used to create a duck density map and to visualize the spatial distribution of migratory waterfowl staging areas and duck farming sites of studied districts (Figure 1). The district-level administrative shape file was retrieved from freely available DIVA-GIS^2^ (54).

### 2.7. Bayesian phylogenetic analysis of H5N1 viruses

To identify the clade diversity of H5N1 viruses circulating among ducks in Bangladesh, On January 1, 2023, all accessible HA gene sequences of A/H5N1 HPAIs found in Bangladesh from ducks with full-length HA sequences were retrieved from the GISAID Epiflu database (55). The HA sequences of H5N1 from 2007 to 2022, were retrieved from GISAID and NCBI and then the artifacts sequence were removed. A Maximum Clade Credibility (MCC) tree using the Bayesian Markov Chain Monte Carlo approach was generated using the temporal information of the sequence data to estimate the evolution of H5N1 viruses in Bangladesh (BEAST 1.10.4) (56). The uncorrelated lognormal clock model with the Bayesian Skyline tree prior was used with 10 million generations (57). The gamma-distributed rate variation among sites with four rate categories (HKYþG) (58) was used. The sampling frequency was 1,000. We visualized the MCC trees in FigTree v1.4.4.^3^ To identify the phylogenetic relationships of the seven H5N1 viruses sequenced in this study, the maximum likelihood method was used. For each gene segment of HA and NA, we used BLAST best matches to select the relevant sequences. TIM + F + G4 model for HA segments and K3Pu + F + G4 for NA segments was chosen by minimum BIC values using IQ-Tree (59). For each tree, we used 1,000 bootstrap replicates for generating the trees. The maximum likelihood tree was also visualized using Figtree v1.4.4.

## 3. Results

### 3.1. Prevalence of AIV, H5, H9, and A/Untyped in ducks and farming types

**Figure 2:**
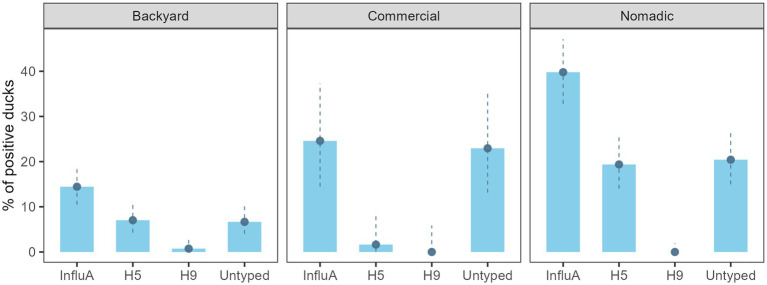
Bangladesh duck level InfluA (M gene), H5, H9, and A/Untyped prevalence with 95% confidence interval during 2019–2021.

AIV prevalence for the overall sampled duck was 24.9% (130/522) (95% CI: 21.3–28.9) (Figure 2). Across the farming system, AIV prevalence was highest in the nomadic duck (76/191) (39.8%; 95% CI: 32.9–47.1) followed by commercial (15/61) (24.6%; 95% CI: 14.5–37.3) and backyard duck (39/270) (14.4%; 95% CI: 10.5–19.2). The H5 prevalence was prominent in nomadic ducks (37/191) (19.4%; 95% CI: 14.0–25.7). There was no H9 subtype found in commercial and nomadic ducks but in two backyard ducks (1.6, 95% CI: 0–8.8) (Figure 2). None of the sample was positive for H7.

**Figure 3:**
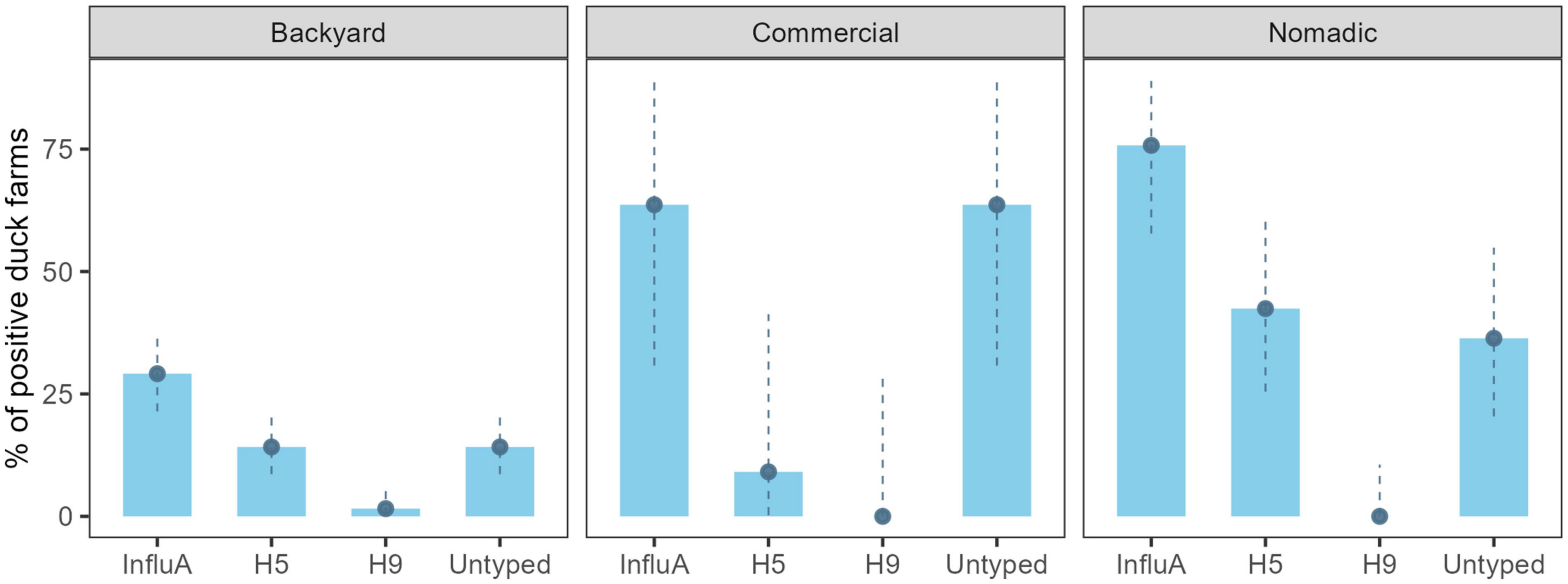
Duck farm level InfluA (M gene), H5, H9, and A/Untyed prevalence with 95% confidence interval in Bangladesh (2019–2021).

On the other hand, AIV prevalence for the overall duck farm was 40.4% (69/171) (95% CI: 33.0–48.1%), backyard farm was 29.1% (37/127) (95% CI: 21.4–37.9), the commercial farm was 63.6% (7/11) (95% CI: 30.8–89.1), and the nomadic farm was 75.8% (25/33) (95% CI: 57.7–88.9) (Figure 3). The H5 subtype was higher (14/33) (42.4%; 95% CI: 25.5–60.8) in the nomadic farming system. In backyard farming, the prevalence of H5 and A/Untyped subtypes on farms were similar (18/127) (14.2%; 95% CI: 8.6–21.5) (Figure 3).

### 3.2. Association of AIV and its subtypes with migratory waterfowl interface

**Figure 4:**
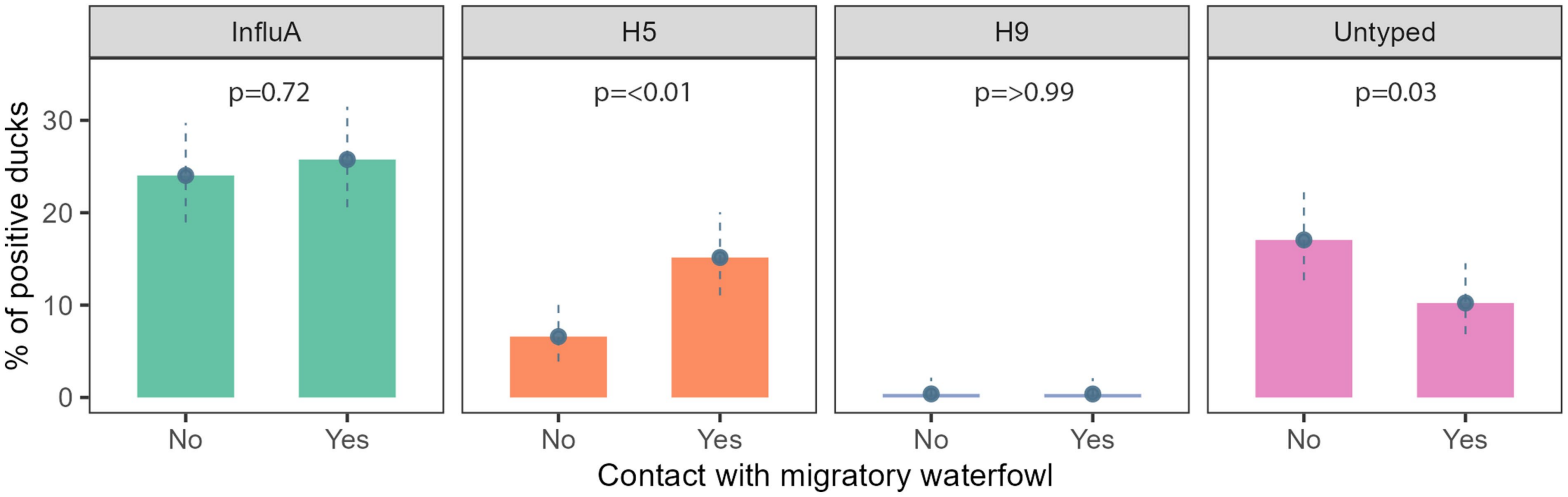
Prevalence of InfluA and its subtypes with migratory waterfowl contact in Bangladesh during 2019–2021.

We found evidence of an association between AIV subtypes and migratory waterfowl present in that area. A/H5 and A/Untyped were significantly associated with the presence of migratory waterfowl (Figure 4).

### 3.3. Risk factor for the circulation of AIV in ducks

We had four variables to check for association with AIV and H5. The farming system, age, and health condition were significantly associated with AIV and H5. Among the farming system, the nomadic system had a higher prevalence for AIV (39.8%; 95% CI: 32.8–47.1%) and H5 (19.4%; 95% CI: 32.8–47.1), whereas backyard and commercial were less positive. Juvenile age group birds were significantly more positive than adults, and sick birds were the most affected by AIV (83.3%; 95% CI: 67.2–93.7) and H5 (80.6%; 95% CI: 64.0–91.8) (Table 1). The sick ducks developed neurological signs including uncoordinated gait circling and torticollis at the terminal stage, digestive symptoms (whitish feces, fecal attached to the plumage and cloaca) and respiratory distress, dilated pupils and followed by death.

**Table 1:** Cross table with chi-square analysis between AIV and bird-level factors of duck Bangladesh isolates (2019–2021).

	A positive (%)	95% CI	Value of *p*	H5 positive (%)	95% CI	Value of *p*
Farming system						
Backyard	39 (14.4)	10.5–19.2	<0.01	19 (7.0)	4.3–10.8	<0.01
Commercial	15 (24.6)	14.5–37.3		1 (1.6)	0–8.8	
Nomadic	76 (39.8)	32.8–47.1		37 (19.4)	14.0–25.7	
Age						
Adult	50 (16.1)	12.2–20.6	0.01	22 (7.1)	4.5–10.5	0.01
Juvenile	80 (37.9)	31.3–44.8		35 (16.6)	11.83–22.31	
Sex						
Female	115 (24.7)	20.8–28.9	0.86	52 (11.2)	8.5–14.4	0.78
Male	15 (26.8)	15.8–40.3		5 (8.9)	3.0–19.6	
Health condition						
Healthy	100 (20.6)	17.1–24.5	<0.01	28 (5.8)	3.9–8.2	<0.01
Sick	30 (83.3)	67.2–93.6		29 (80.6)	64.0–91.8	

Value of *p* < 0.05; statistically significant.

In the multivariable logistic regression model, we found three variables as significant risk factors for AIV and one risk factor for A/H5. The nomadic farming system had 2.39 times (95% CI: 1.45–3.93) higher odds of affecting AIV than backyard farming (*p* = 0.01). Compared to adults, juvenile ducks had 2.22 times (95% CI: 1.37–3.61) odds of having AIV (*p* = 0.01). The AIV detection in sick ducks (the ducks displayed dilated pupils and white feces remained on the plumage surrounding the cloaca and neurologic symptoms include an uncoordinated gait, tremors, and torticollis) was 11.59 times (95% CI: 4.82–32.44) more likely (*p* < 0.01) than healthy birds (Table 2). We found health conditions to be a significant risk factor for H5. The odds of H5 detection in sick birds were 46.5 times (95% CI: 18.7–130.3) more likely than healthy ones (*p* < 0.01) (Table 2).

**Table 2:** Risk factors of AIV and A/H5 circulation in individual ducks from the different production systems in Bangladesh (2019–2021).

	A (M gene)		A/H5	
	AOR (95% CI)	Value of *p*	AOR (95% CI)	Value of *p*
Farming system				
Backyard	Reference		Reference	
Commercial	1.3 (0.6–2.7)	0.51	0.2 (0–1.1)	0.14
Nomadic	2.4 (1.5–3.9)	<0.01	1.3 (0.6–2.7)	0.56
Age				
Adult	Reference		Reference	
Juvenile	2.2 (1.4–3.6)	<0.01	1.7 (0.8–3.5)	0.18
Health condition				
Apparently healthy	Reference		Reference	
Sick	11.6 (4.8–32.4)	<0.01	46.5 (18.7–130.3)	<0.01

Value of *p* < 0.05; statistically significant.

### 3.4. Bayesian phylogenetic analysis of the evolution of H5N1 clades in Bangladeshi ducks

**Figure 5:**
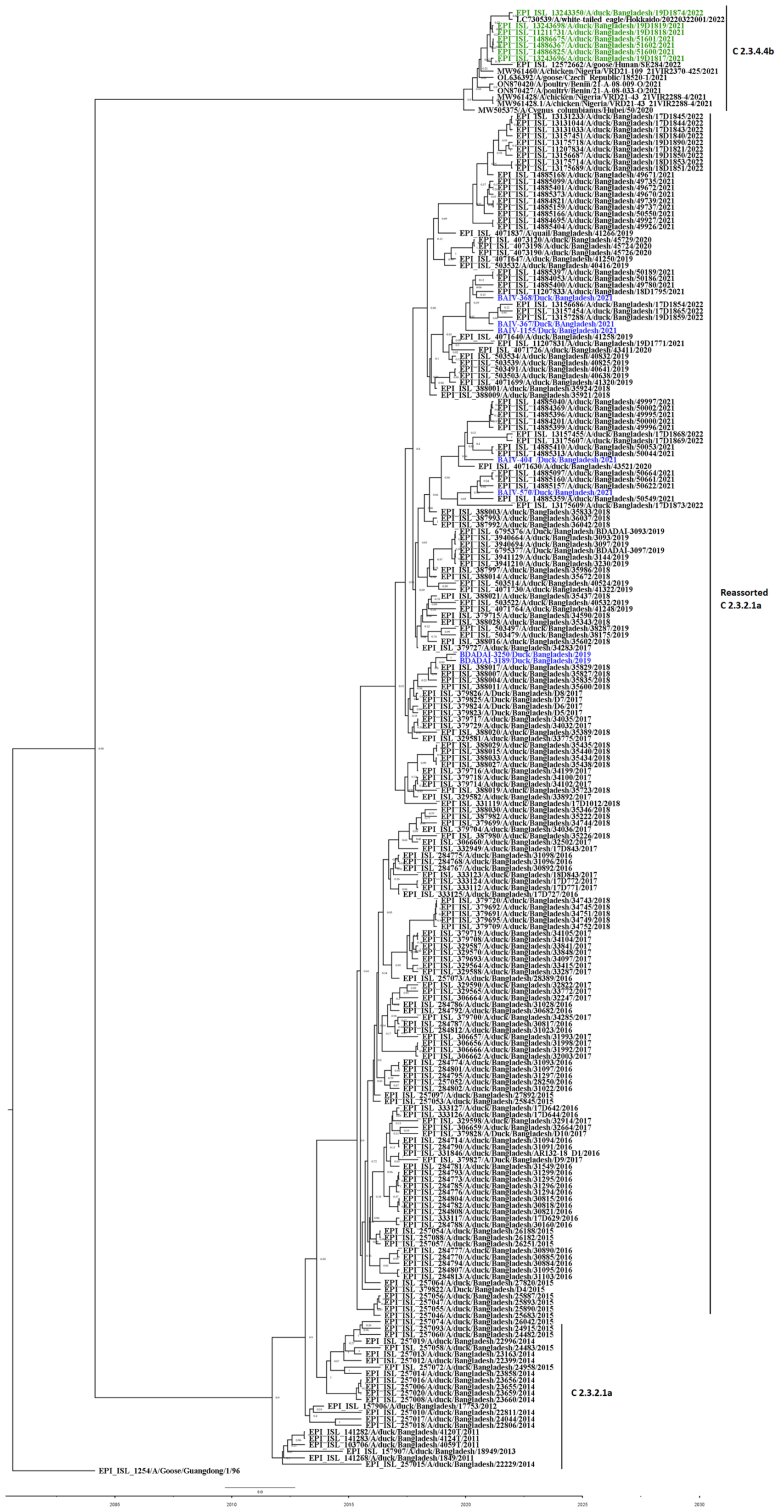
Bayesian phylogenetic tree of H5 HA viruses of diverse clades in Duck in Bangladesh. Taxon labels with blue color indicate the viruses found in ducks under this study, and taxon labels with green color indicate those reassorted H5N1 viruses detected as clade 2.3.4.4b.

**Figure 6:**
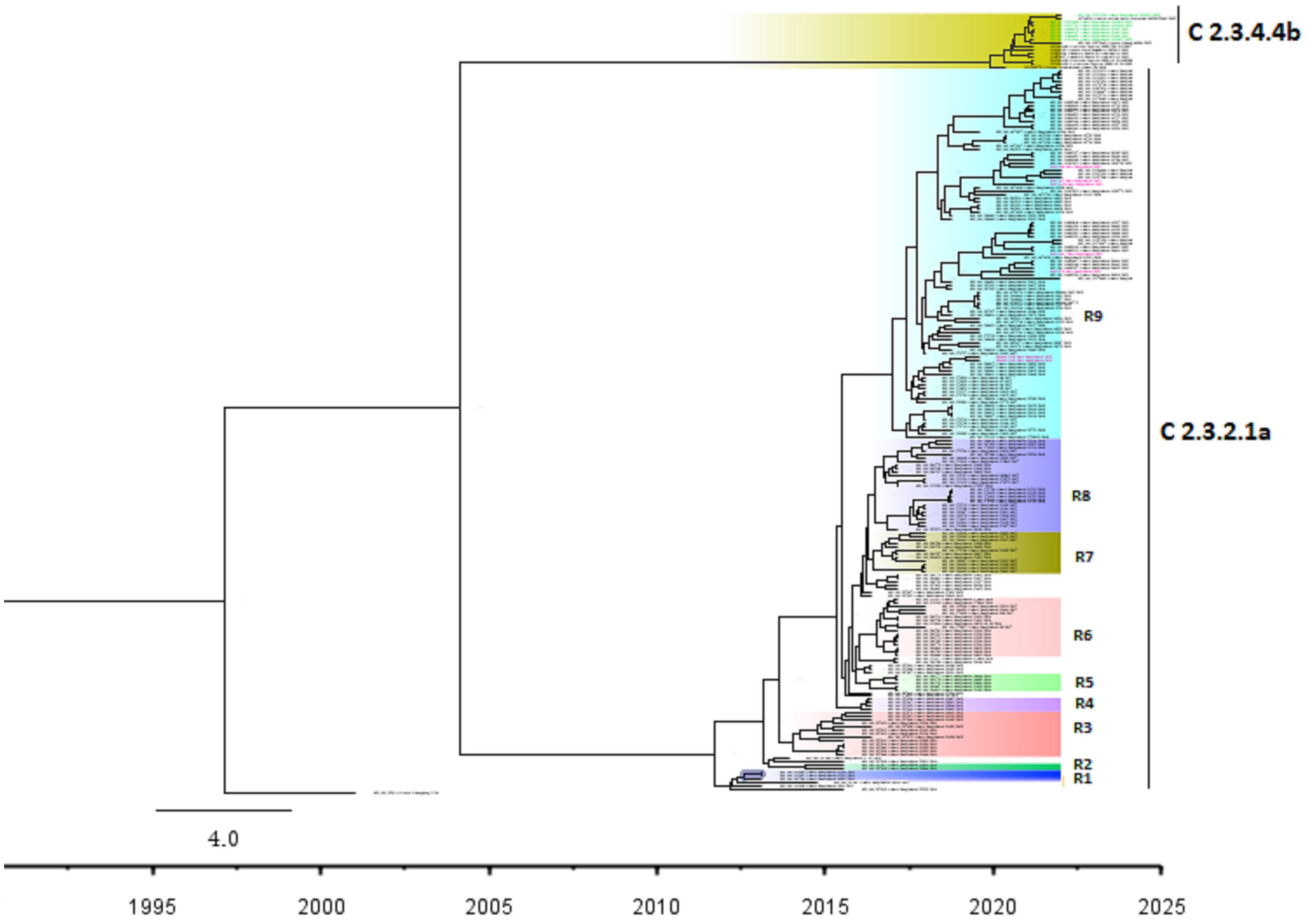
Bayesian phylogenetic tree of H5 HA viruses of diverse clades along with subgroups based on >90% posterior probability in Duck in Bangladesh. Taxon labels with red color indicate the viruses found in ducks under this study, and taxon labels with green color indicate those reassorted H5N1 viruses detected as clade 2.3.4.4b. Each colored box indicates a subgroup.

The Bayesian phylogenetic tree (Figure 5) indicates that clade 2.3.2.1a has been circulating in ducks in Bangladesh since 2011. In 2015, the novel reassortant of the clade 2.3.2.1a H5N1 virus was discovered in ducks (Figure 5). The majority of H5N1 viruses detected in waterfowl are novel reassortant of clade 2.3.2.1a. Seven H5N1 sequences were identified as belonging to the emerging panzootic clade 2.3.4.4b (Figure 5). Sequences from this emerging clade clustered with white-tailed eagles from Japan (Hokkaido), geese from China (Hunan), and chickens and ducks from Africa (Nigeria and Benin). These sequences share a similarity of between 98.65 and 98.97% with H5N1 viruses of clade 2.3.4.4b from Japan and a similarity of 99.30% with viruses from China. This new clade may have been introduced to Bangladesh by the end of 2020 (Figure 5). Figure 6 shows clade 2.3.2.1a has been silently evolving among ducks, and based on posterior probability >90%, and that the clade has formed at least nine subgroups among ducks in Bangladesh. Currently, only subclade R9 of clade 2.3.2.1a is circulating in ducks in Bangladesh.

### 3.5. Maximum likelihood phylogenetic analysis of HA and NA sequences of H5N1 viruses isolated in ducks in Bangladesh

**Figure 7:**
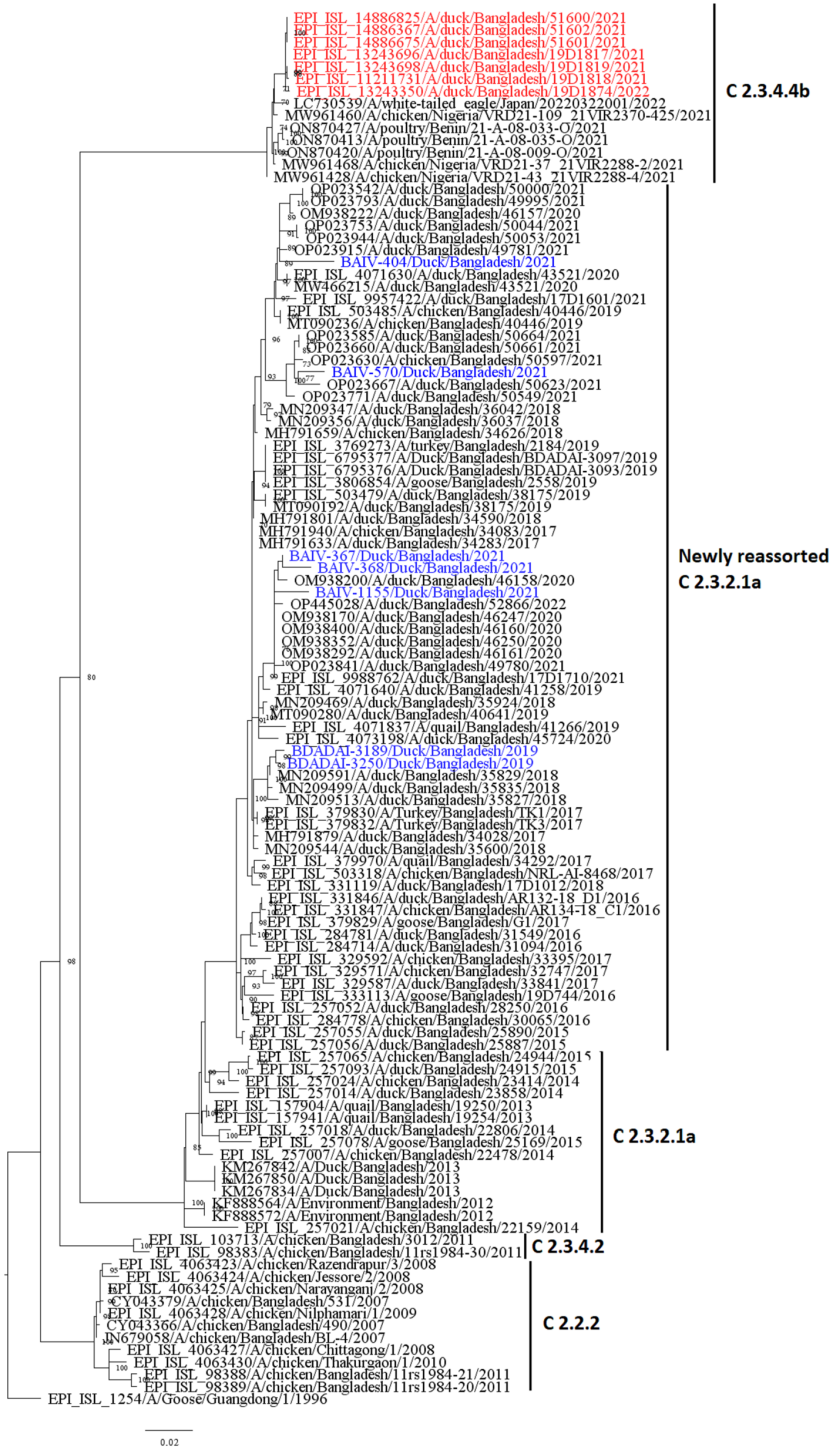
Maximum likelihood tree of HA sequences of H5N1 viruses in Bangladesh. Taxon labels with blue color indicate the viruses found in ducks under this study, and taxon labels with red color indicate those reassorted H5N1 viruses detected as clade 2.3.4.4b.

**Figure 8:**
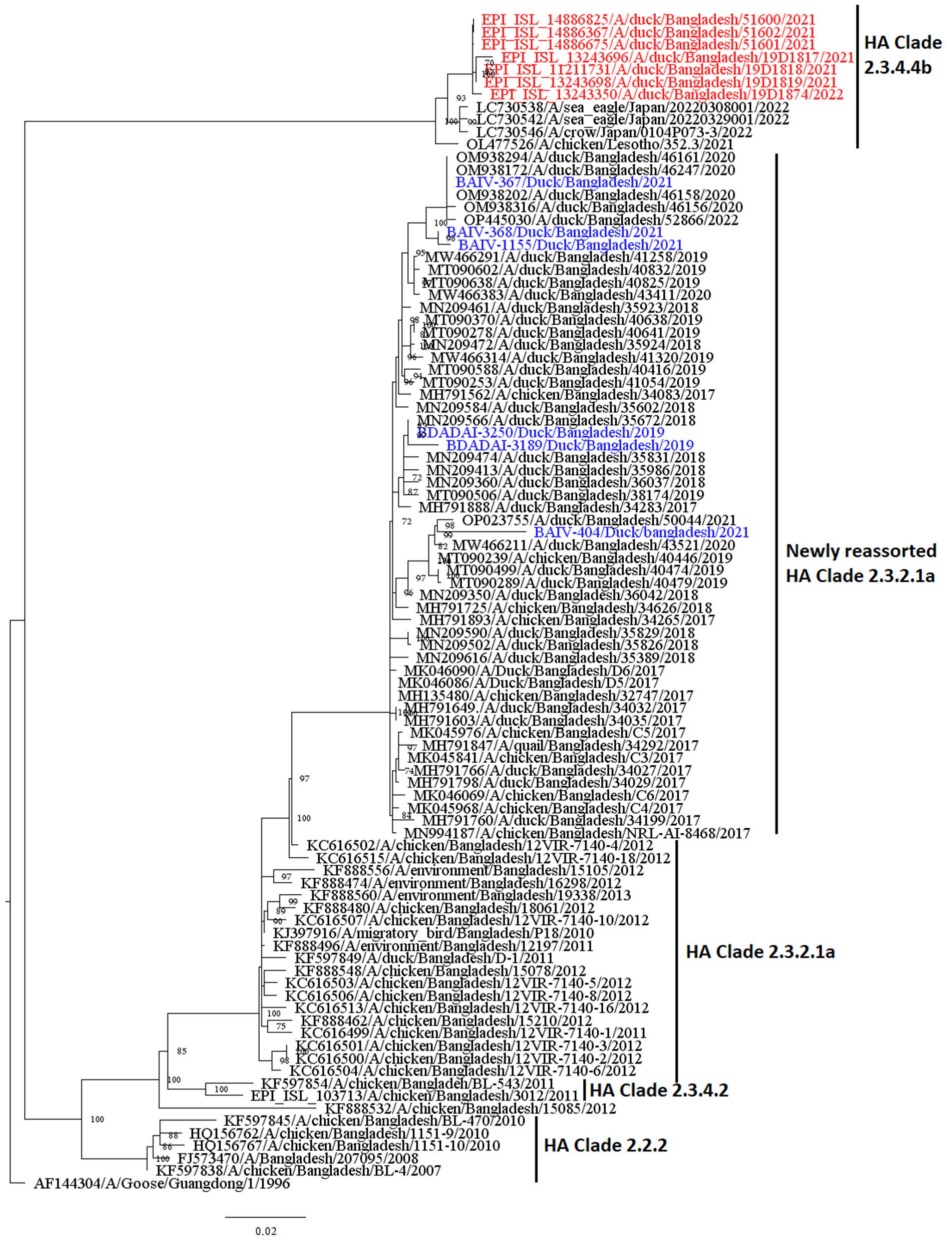
Maximum likelihood tree of 6 NA sequences of H5N1 viruses. Taxon labels with blue color indicate the viruses found in ducks under this study, and taxon labels with red color indicate those reassorted H5N1 viruses detected as HA clade 2.3.4.4b.

Figures 7, 8 present the maximum likelihood phylogenetic trees of HA and NA gene segments of H5N1 viruses sequenced in this study. Five H5N1 viruses were detected in 2021 and the two viruses in 2019 belonged to the newly reassorted clade 2.3.2.1a. However, they clustered in different groups within this clade. The two virus sequences from 2019 have clustered together. The maximum likelihood tree of HA also shows that the viruses we detected in duck hosts are similar to those found in chickens. BAIV-570 and BAIV-404 have clustered within a group with virus sequences obtained from chicken (Bootstrap value>95%).

## 4. Discussion

### 4.1. Prevalence and risk factors of AIV and subtypes in the different duck production systems

The high prevalence of AIV with the H5N1 subtype in nomadic ducks compared to backyard and commercial ducks was consistent with the other study conducted by Khatun et al. (60), reported a higher prevalence of AIV in ducks reared in the hoar (wetland) region where the nomadic system is prevalent. This is because of the higher density of migratory birds in the hoar area, with a possible most increased interaction between the native duck and migratory bird species (61). Previous studies in Bangladesh detected AIV with H5N1 in both domestic and migratory ducks in wetland areas where domestic ducks and migratory birds shared the same feeding habitats in wetlands (62, 63).

Furthermore, the farm-level prevalence of AIV was also higher in nomadic ducks, supported by Hasan et al. (61) because the grazing land ecosystem is a critical factor for the circulation and spread of AIV. Concerning risk factors, there is a significant association among different farming systems, which is also supported by Henning et al. (64) reported that the birds that used to scavenge are most frequently affected. Juvenile ducks were mostly affected by both AIV M-gene and H5 subtype, which was supported by Strurm-Ramirez et al. (65). Our study revealed that the farming system significantly impacts the presence of AIV in ducks. The odds of AIV have been observed to be greater in ducks from nomadic farms than in backyard ducks. As low-lying areas with vast bodies of water are a favorable environment for raising nomadic ducks, and they have more interaction with migrating waterfowl than a backyard or commercial ducks, previous studies have shown that nomadic ducks are more susceptible to the AIV (66, 67). Our study also showed that juvenile ducks are more likely to be infected by AIV than adult ducks. A study in Canada also reported a higher detection rate of AIV in juvenile ducks than in adults (68). Adult birds presumably have acquired immunity or an enhanced immune response, but juvenile birds are immunologically more naive, rendering them more vulnerable to viral infection than adult birds (69).

Our study also showed that detecting AIV and A/H5 is higher in sick ducks than in apparently healthy ducks. Previous studies in Bangladesh and other countries have reported similar results for ducks and poultry birds (2, 70–73). Even though ducks can secrete large quantities of a deadly virus without manifesting any outward signs of disease, H5N1 can cause the birds to have breathing difficulties such as gaping (mouth breathing), nasal snicking (coughing sound), sneezing, gurgling, or rattling. Since AIV causes bird sickness, the detection rate of AIV and H5 is higher in sick ducks (74–76).

### 4.2. Phylodynamic of multiple clades of H5N1 viruses in duck farms

According to our study, multiple H5N1 virus clades are spreading in Bangladesh. Our study shows that two clades of H5N1 viruses are now circulating among ducks in Bangladesh. These two clades, 2.3.2.1a and 2.3.4.4b, of H5N1 viruses in ducks, have also been detected in nearby countries such as India and China (77, 78). Our study shows that clade 2.3.2.1a has been detected in ducks since 2011 and has become endemic in ducks in Bangladesh. Similar to our findings, other studies reported that this clade reassorted, resulting in a new subclade in 2015 (79). According to Barman et al. (32), this novel reassortant clade 2.3.2.1a virus emerged in Bangladesh via reassortment with LPAI viruses transmitted by migrating birds. Despite vaccination of commercial chicken farms in Bangladesh, Clade 2.3.2.1a HPAI has caused ongoing outbreaks in Bangladesh since 2011. Our study also shows that viruses of clade 2.3.2.1a have created at least nine subgroups within ducks based on >90% posterior probability. This suggests that the virus of this clade is silently evolving, and ducks may play an important role in the emergence of new clades in Bangladesh. Prior studies have also shown that clade 2.3.2.1a HPAIs are circulating in LBMs and domestic ducks in Bangladesh, where they play an important role in the maintenance and development of new reassortant viruses (25, 80).

The findings of the phylogenetic study also revealed that clade 2.3.4.4b was introduced to Bangladesh by the end of 2020. Cui et al. (33) also reported that one H5N1 virus from Bangladesh clustered with Chinese viruses within the 2.3.4.4b clade. Since its emergence in the Netherlands in October 2020, H5N1 viruses with the clade 2.3.4.4b HA gene have spread to several countries in Europe, Africa, Asia, and North and South America (81, 82). On the other hand, in China, Between September 2021 and March 2022, H5N1 viruses bearing the HA clade 2.3.4.4b were discovered in wild birds and domestic poultry (33). Our study shows that seven viruses clustering within the 2.3.4.4b clade have similarities with viruses from Japan and China. So, it might be possible that migratory birds of the Central Asian flyway may influence the transmission of this novel clade in Bangladesh. Though the H5N1 viruses with clade 2.3.4.4b have only been detected in Ducks in Bangladesh, this clade has been detected in wild birds and domestic Anseriformes and Galliformes in other countries (83–85). On the other hand, this clade has also caused outbreaks in minks in Spain (86). More than 50 thousand mink were killed and their carcasses destroyed, and it was assumed that wild birds may have played a major role in the transmission of the virus (34). This virus has also been detected in harbor porpoises in Sweden (35) and dolphins, sea lions, sanderlings, pelicans, and cormorants in Peru (36), along with 8 human cases since 2022 (37). It is extremely alarming because the H5N1 virus is known to spread poorly among mammals; humans almost exclusively contract it from infected birds. However, it has since been established that the 2.3.4.4b outbreak in minks spread throughout a tightly-knit mammalian population (87). Given that the virus has already been introduced to Bangladesh, it is likely that this clade may also spread to chickens and other poultry through ducks and wild birds. As a result, there is a danger of transmission among humans as well as the possibility of a pandemic. We recommend carrying out a thorough risk analysis so that decision-makers may fully comprehend the risks connected to AIV and H5N1 outbreaks, the possible effects of the epidemic, and the steps that can be done to prevent or mitigate the disease’s transmission.

## 5. Conclusion

This study demonstrates that H5N1 circulating in all three duck farming production systems and nomadic farms poses a higher risk of AIV infection than those from residential or commercial farms. Age and health of ducks influence the risk of AIV and H5N1 infection in populations of ducks. Clades 2.3.2.1a and 2.3.4.4b of H5N1 are circulating in Bangladeshi waterfowl. The duck farmer should receive appropriate training to enhance farm biosecurity practices in order to prevent the spread of AIV. Enhanced AIV surveillance is necessary for both domestic and migratory waterfowl, with a focus on Anseriformes production systems, to analyze the genetic diversity of H5N1 viruses and to determine the evolution of the virus at high-risk interfaces between domestic ducks and migratory birds.

## Data availability statement

The datasets presented in this study can be found in online repositories. The names of the repository/repositories and accession number(s) can be found in the article/supplementary material.

## Ethics statement

The studies involving human participants were reviewed and approved by the Ethics Committee (EC) (Protocol: CVASU/Dir(R&E) EC/2020/191/7) of the Chattogram Veterinary and Animal Sciences University. The participants provided their written informed consent to participate in this study. The animal study was reviewed and approved by the Chattogram Veterinary and Animal Sciences University-Animal Experimentation Ethics Committee (protocol: CVASU/Dir(R&E) EC/2020/191/7). Written informed consent was obtained from the owners for the participation of their animals in this study.

## Author contributions

AI: conceptualization. AI, MdH, and SI: methodology. AI, MdH, SI, and MAS: field investigation. AI, MAS, and MI: validation.AI, EA, and MI: software and formal analysis. AI and EA:visualization and writing—original draft. AI, EA, MMH, and MI: data curation. MEH, MM, and MZR: laboratory analysis.AI, MAS, MI, and MMH: writing—review, and editing. TS and MZR: supervision. MMH, TS, and MZR: project administration and funding acquisition. All authors contributed to the article and approved the submitted version. All authors have read and approved the final version of the manuscript.

## Funding

The sample collection was supported by the University Grant Commission (UGC) of Bangladesh through Chattogram Veterinary and Animal Sciences University (CVASU), grant number UGC/CVASU#06, and the United States Agency for International Development (USAID) Emerging Pandemic Threats PREDICT project (cooperative agreement number: AID-OAA-A-14-00102) through EcoHealth Alliance. We thank the governments of Bangladesh, Canada, Sweden, and the United Kingdom for providing core/unrestricted support to icddr, b. The team was partially supported by the Intramural Research Program of the National Institute of Allergy and Infectious Diseases (NIAID) National Institutes of Health (NIH) (U01AI153420).

## Conflict of interest

The authors declare that the research was conducted in the absence of any commercial or financial relationships that could be construed as a potential conflict of interest.

## Publisher’s note

All claims expressed in this article are solely those of the authors and do not necessarily represent those of their affiliated organizations, or those of the publisher, the editors and the reviewers. Any product that may be evaluated in this article, or claim that may be made by its manufacturer, is not guaranteed or endorsed by the publisher.

## Footnotes

^1^ https://www.arcgis.com

^2^ https://www.diva-gis.org/gdata

^3^ http://tree.bio.ed.ac.uk/software/figtree/
